# Infection à EBV compliquée d'un syndrome d'activation macrophagique

**DOI:** 10.11604/pamj.2015.20.255.5903

**Published:** 2015-03-17

**Authors:** Madiha Mahfoudhi, Sami Turki

**Affiliations:** 1 Service de Médecine Interne A, Hôpital Charles Nicolle, Tunis, Tunisie

**Keywords:** Infection à EBV, hémophagocytose, hépatite fulminante, EBV infection, hemophagocytosis, fulminant hepatitis

## Image in medicine

Le syndrome d'activation macrophagique est une complication rare qui peut aggraver le pronostic d'une infection, une néoplasie ou une maladie auto-immune. Il complique rarement une infection à EBV et constitue un élément de très mauvais pronostic. Patient âgé de 18 ans hospitalisé pour une fièvre. Il avait des adénopathies cervicales et une hépatomégalie. L'examen de la gorge, l'auscultation cardiaque et pulmonaire ainsi que l'examen neurologique étaient sans anomalies. La radiographie du thorax était normale. L’échographie abdominale et la TDM thoraco-abdomino-pelvienne ont objectivé une hépatomégalie homogène et des adénopathies centimétriques péri-aortiques. A la biologie, il avait une hyperferritinémie, une hypertriglycéridémie, un syndrome inflammatoire biologique, une pancytopénie avec une leucopénie à 1700 éléments /mm3, une anémie normochrome normocytaire à 7g/dl et une thrombopénie à 70000 éléments /mm3. En outre, il avait une cytolyse hépatique à 5 fois la normale. Les infections virales de type hépatite virale C, hépatite virale B, HIV, herpes simplex virus, varicelle zona virus, cytomégalovirus et parvovirus B19 étaient éliminées. La sérologie EBV était positive avec des taux d'Ig G et d'Ig M élevés. La PCR d'EBV était positive. Une hémophagocytose compliquant cette infection à EBV a été confirmée par le myélogramme. L’évolution était marquée par la survenue d'une hépatite fulminante avec apparition de signes d'insuffisance hépatocellulaire. Une insuffisance rénale aigue a compliqué le tableau avec une créatinémie à 910 µmol/l. Un traitement par étoposide était prévu mais le patient était décédé dans tableau de défaillance multiviscérale.

**Figure 1:**
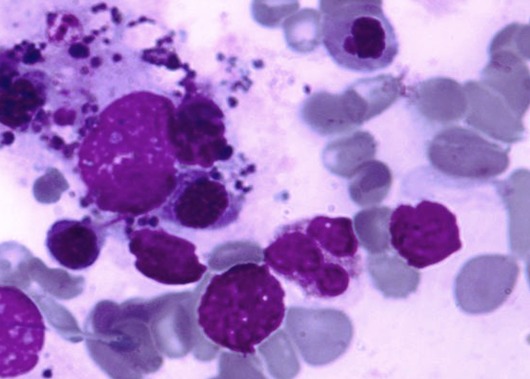
Myélogramme: hémophagocytose

